# Taxonomy, ecology, and relevance to food safety of the genus *Listeria* with a particular consideration of new *Listeria* species described between 2010 and 2022

**DOI:** 10.1128/mbio.00938-23

**Published:** 2023-12-21

**Authors:** Renato H. Orsi, Jingqiu Liao, Catharine R. Carlin, Martin Wiedmann

**Affiliations:** 1 Department of Food Science, Cornell University, Ithaca, New York, USA; 2 Department of Civil and Environmental Engineering, Virginia Tech, Blacksburg, Virginia, USA; 3 Mérieux NutriSciences, Crete, Illinois, USA; Albert Einstein College of Medicine, Bronx, New York, USA

**Keywords:** *Listeria*, species, *Murraya*, *Paenilisteria*, *Mesolisteria*, detection methods, identification methods, food safety, rapid methods

## Abstract

Since 2010, the genus *Listeria* has had the addition of 22 new species that more than tripled the number of species identified until 2010. Sixteen of these 22 new species are distantly related to the type species, *Listeria monocytogenes*, and several of these present phenotypes that distinguish them from classical *Listeria* species (*L. monocytogenes*, *Listeria innocua*, *Listeria ivanovii, Listeria seeligeri*, *Listeria welshimeri*, and *Listeria grayi*). These 22 newly described species also show that *Listeria* is more genetically diverse than previously estimated. While future studies and surveys are needed to clarify the distribution of these species, at least some of these species may not be widely spread, while other species may be frequently found spread to human-related settings (e.g., farms and processing facilities), and others may be adapted to specific environmental habitats. Here, we review the taxonomic, phylogenetic, and ecological characteristics of these new *Listeria* species identified since 2010 and re-iterate the suggestion of re-classification of some species into three new genera: *Murraya*, *Mesolisteria*, and *Paenilisteria*. We also provide a review of current detection issues and the relevance to food safety related to the identification of these new species. For example, several new non-pathogenic species could be misidentified as the pathogen *L. monocytogenes*, based on methods that do not target *L. monocytogenes*-specific virulence genes/factors, leading to unnecessary product recalls. Moreover, eight species in the proposed new genus *Mesolisteria* are not good indicators of environmental conditions that could allow *L. monocytogenes* to grow since *Mesolisteria* species are unable to grow at low temperatures.

## INTRODUCTION

*Listeria* is one of the only two genera within the family *Listeriaceae*; the other genus in the family is *Brochothrix*. Until 2010, the genus *Listeria* included only six species (i.e., *Listeria monocytogenes*, *Listeria seeligeri*, *Listeria welshimeri*, *Listeria innocua*, *Listeria ivanovii*, and *Listeria grayi*; hereafter referred to as “classic” *Listeria* species). *Listeria* have been commonly described as small Gram-positive, nonspore forming, noncapsulated, motile bacilli (1). Conventional *Listeria* identification approaches include Gram staining, observation of motility, and biochemical reactions, such as catalase and D-glucose fermentation (2). Two *Listeria* species are considered pathogenic, *L. monocytogenes* and *L. ivanovii* (3), with *L. ivanovii* generally causing illness only in ruminants (4), and *L. monocytogenes* causing illness in humans, in addition to other animals. *L. monocytogenes* is almost exclusively transmitted to humans through foods, with an estimated 99% of infections being foodborne (5). Although *L. monocytogenes* infections are relatively rare (estimated 3,175 cases per year globally [6]), the high hospitalization rate (94% [5]) and mortality rate (15.9–22% [5, 6]) make *L. monocytogenes* a major bacterial foodborne hazard.

In 2010, two new species, *Listeria marthii* (7) and *Listeria rocourtiae* (8), were described. *L. marthii* showed most of the phenotypic characteristics of the classic *Listeria* species and was shown to be phylogenetically closely related to *L. monocytogenes* (7). *L. rocourtiae*, however, is non-motile and was found to be phylogenetically more closely related to *L. grayi* than to the other classic *Listeria* species (8). Since 2013, 20 new *Listeria* species have been described (an average of two new species per year), with many of them showing at least some phenotypic characteristics incongruent with their classification within the genus *Listeria* (e.g., lack of ability to grow at 4°C for at least 8 species and lack of motility for at least 15 species). In 2013, *Listeria weihenstephanensis* (9) and *Listeria fleischmannii* (10) were described, followed by *Listeria floridensis*, *Listeria aquatica*, *Listeria cornellensis*, *Listeria riparia*, and *Listeria grandensis* in 2014 (11), *Listeria booriae* and *Listeria newyorkensis* in 2015 (12), *Listeria costaricensis* (13) and *Listeria goaensis* (14) in 2018, *Listeria thailandensis* in 2019 (15), *Listeria valentina* in 2020 (16), *Listeria portnoyi*, *Listeria rustica*, *Listeria farberi*, *Listeria immobilis*, and *Listeria cossartiae* in 2021 (17), and *Listeria ilorinensis* (18) and *Listeria swaminathanii* (19) in 2022 (20–22). Here, we provide a review of the phylogenetic, phenotypic, and ecological characteristics of the newly described *Listeria s*pecies, with a special focus on those described between 2010 and 2022. We also show the practical impact of the sudden increase in the number of species within the genus, and the implications for the food industry, which uses testing for *Listeria* spp. in the processing environment to facilitate control of the foodborne pathogen *L. monocytogenes* (since *L. monocytogenes* and *Listeria* spp. were considered to thrive under similar conditions, such as low temperatures).

## PHYLOGENY OF THE CURRENT GENUS LISTERIA

**Fig 1:**
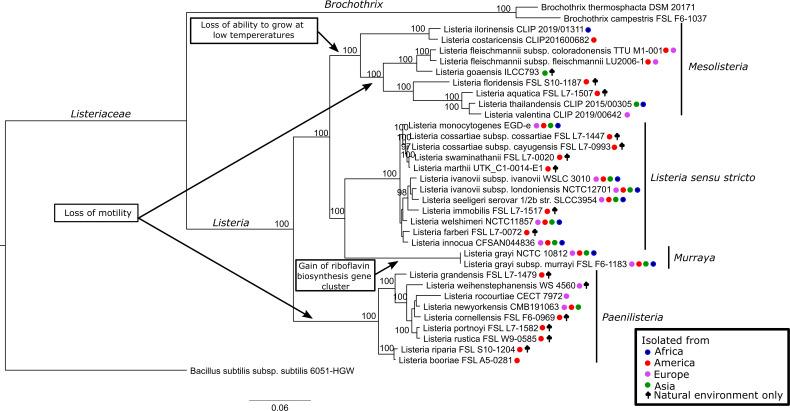
Maximum likelihood phylogenetic tree based on the concatenated alignment of 24 core genes identified using Panaroo (24). The tree was constructed with RAxML (25) using the GTRCAT model and 1,000 bootstraps. Bootstrap values above 75% are shown next to their respective nodes. Circles after the names are color-coded according to the location where each species has been identified to date. Isolates only identified from natural environments are also indicated. Suggested new genera names (i.e., *Murraya*, *Mesolisteria*, and *Paenilisteria*) for the three *Listeria sensu lato* clades are shown at the right of each clade. The tree was rooted at the *Bacillus subtilis* subsp. *subtilis* strain 6051-HGW branch. Major phenotypic changes are indicated by arrows pointing to the branches where the changes most likely occurred. For “gain of riboflavin biosynthesis gene cluster” and “loss of ability to grow at low temperatures,” gain and loss, respectively, are the most parsimonious explanation for the patterns of phenotypes observed among different clades. For the event marked as “Loss of motility,” however, either two gain events or two loss events are equally parsimonious.

The family *Listeriaceae* is composed of two genera, *Listeria* and *Brochothrix*, both of which are monophyletic (Fig. 1). The genus *Listeria* has been classified into two groups named: *sensu stricto* and *sensu lato. Listeria sensu stricto* refers to the 10 current species (*L. monocytogenes*, *L. seeligeri*, *L. welshimeri*, *L. innocua*, *L. ivanovii*, *L. marthii*, *L. farberi*, *L. immobilis*, *L. cossartiae*, and *L. swaminathanii*) closely related to, and including, *L. monocytogenes*, the first species identified in 1924 (23). Although *Listeria sensu lato* originally referred to all species within the genus *Listeria*, recent publications have used the term “*sensu lato*” to refer specifically to those *Listeria* species that are less phylogenetically related to *L. monocytogenes* (i.e., those species not in the *Listeria sensu stricto* group). *Listeria sensu lato* currently includes 18 species (*L. grayi*, *L. fleischmannii*, *L. floridensis*, *L. aquatica*, *L. valentina*, *L. thailandensis*, *L. goaensis*, *L. ilorinensis*, *L. costaricensis*, *L. rustica*, *L. portnoyi, L. cornellensis*, *L. newyorkensis*, *L. rocourtiae*, *L. weihenstephanensis*, *L. grandensis*, *L. booriae*, and *L. riparia*). Although *Listeria sensu stricto* forms a monophyletic group (i.e., a clade), *Listeria sensu lato* seems to be paraphyletic, as the most recent common ancestor (most recent common ancestor (MRCA), in this case, the ancestor of all *Listeria* species) of *Listeria sensu lato* is also an ancestor of all *Listeria sensu stricto* species (Fig. 1) (17–19). Nevertheless, within the paraphyletic *Listeria sensu lato*, there are three major clades. One clade contains only one species, *L. grayi*, which includes two subspecies, *L. grayi* subsp. *grayi* and *L. grayi* subsp. *murrayi*; the two other clades contain eight and nine species.

Within *Listeria sensu stricto*, phylogenetic analyses show that *L. monocytogenes* share an MRCA with *L. cossartiae*, *L. marthii*, and *L. swaminathanii* (Fig. 1) (19, 26); the species *L. ivanovii*, *L. seeligeri*, and *L. immobilis* are the most distantly related to *L. monocytogenes* within *Listeria sensu stricto. Listeria sensu stricto* also seems to have a much lower genetic diversity as compared to each of the two clades within *Listeria sensu lato* that have more than one species (Fig. 1). *Listeria sensu stricto* shows an average nucleotide identity (ANI) using Blast (ANIb) between 85% and 95% among most *Listeria sensu stricto* species, compared to an ANIb below 85% among most species within these two *Listeria sensu lato* clades (19), suggesting that either *Listeria sensu stricto* represents a more recent clade within the genus *Listeria*, or the species within *Listeria sensu stricto* may have evolved much slower than the species within *Listeria sensu lato*.

Given its high genetic and phenotypic divergence from *Listeria sensu stricto* (ANIb of 70%–73% between *Listeria sensu lato* and *Listeria sensu stricto* species [19]; see phenotypic characteristics below for a description of the phenotypes that differentiate the *Listeria sensu lato* species from the *Listeria sensu stricto* species), the three clades of *Listeria sensu lato* have been previously proposed to represent three different genera (27, 28). The *L. grayi* clade was suggested to represent the proposed genus *Murraya* (27, 28), while the two other clades were suggested to represent the proposed genera *Mesolisteria* (currently including the species *L. fleischmannii*, *L. floridensis*, *L. aquatica*, *L. valentina*, *L. thailandensis*, *L. goaensis*, *L. ilorinensis*, and *L. costaricensis*) and *Paenilisteria* (currently including the species *L. rustica*, *L. portnoyi, L. cornellensis*, *L. newyorkensis*, *L. rocourtiae*, *L. weihenstephanensis*, *L. grandensis*, *L. booriae*, and *L. riparia*).

While *Mesolisteria* and *Paenilisteria* would represent new genera with several species already belonging to them, the genus *Murraya* would initially contain a single species, the current *L. grayi*. The fact that *L. grayi* does not have any closely related species identified so far is intriguing. It is possible that limited divergence and speciation in the ancestral lineage of *L. grayi* occurred, and no *L. grayi*-related species existed, or putative *L. grayi*-related species existed at some time but went extinct. Other hypotheses related to current methods used for detection and identification of *Listeria* isolates could also explain the lack of *L. grayi*-related species to date. For example, although *L. grayi* seems to grow well in the FDA BAM enrichment broth BLEB (buffered *Listeria* enrichment broth), it has been shown that both subspecies of *L. grayi* have reduced growth in some selective media {Fraser, MOPS-BLEB [3-(N-morpholino) propanesulfonic acid–buffered *Listeria* enrichment broth], and UVM (University of Vermont medium)}, typically used to enrich for *Listeria* (29), while *L. grayi* subsp. *murrayi* also shows reduced growth in brain heart infusion (BHI) broth at 30°C when compared to all other *Listeria* species (29). Hence, it is possible that other *L. grayi*-like species have not been identified because they do not grow well in at least some of the selective and enrichment media that are and typically have been used for *Listeria*. Moreover, both *L. grayi* subspecies showed atypical colony morphology on ALOA (agar *Listeria*, according to Ottaviani and Agosti) agar plates, while *L. grayi* subsp. *murrayi* also showed atypical morphology on MOX (modified Oxford) agar plates, where very small colonies (suggesting partial inhibition) were observed (29). Therefore, *L. grayi*-like species may also not have been identified due to atypical morphologies on selective and differential agar plates. Given these difficulties in obtaining *L. grayi* using standard methods used to isolate *Listeria* from samples, it is possible that future metagenomics studies using DNA directly extracted from samples and long-read sequencing will be able to initially identify new species closely related to *L. grayi*; subsequent focused isolation efforts could then be pursued to isolate these species to facilitate formal species descriptions.

## PHENOTYPIC CHARACTERISTICS

All *Listeria sensu stricto* species present most of the characteristics typical of *Listeria*, including being Gram-positive short rods, catalase positive, showing a positive result for the Voges-Proskauer test (which assesses the production of acetoin from glucose fermentation), oxidase negative, unable to reduce nitrite or nitrate, able to ferment D-arabitol, methyl-α-D-glucopyranoside, D-maltose, D-lactose, unable to ferment inositol, D-mannitol, and L-arabinose, and able to grow at 4°C and 41°C. A few *sensu stricto* species, however, lack specific characteristics that are common to most *sensu stricto* species (Table S1). For example, *L. immobilis* is the only *sensu stricto* species to date that is non-motile at all temperatures (due to the lack of the flagellar gene operons in this species), and *L. welshimeri* is the only *sensu stricto* species that can ferment D-tagatose. Moreover, the *L. swaminathanii* type strain is catalase negative. However, two other isolates of this new species were shown to be catalase positive (26), suggesting that the catalase phenotype is variable in *L. swaminathanii*, similarly to what has been documented for *L. monocytogenes* (30–33). Although five new *Listeria sensu stricto* species have been described since 2010, none of them are pathogenic; *L. monocytogenes* and *L. ivanovii* are still considered the only pathogenic species. Both *L. monocytogenes* and *L. ivanovii* show β-hemolysis and phosphoinositide phospholipase C (PI-PLC) activity (*L. seeligeri* is also β-hemolytic but shows no PI-PLC activity and is not considered pathogenic).

As mentioned above, *Listeria sensu lato* species are not genetically and phenotypically congruent, and it has been previously suggested that the species within *Listeria sensu lato* should be re-classified into three new genera (27). *L. grayi*, which has been proposed to be re-classified as *Murraya grayi*, is the only *Listeria sensu lato* species that exhibit motility consistent with the classic *Listeria* spp. (i.e., motile at 20–25°C and non-motile at 35–37°C) and is positive for D-arylamidase activity. In addition, *L. grayi* can ferment D-mannitol while none of the *Listeria sensu stricto* species are able to do this.

In 2016, when Orsi et al. suggested the re-classification of *Listeria sensu lato* into three new genera, the genus name *Mesolisteria* was suggested for three phylogenetically related species identified at the time (i.e., *L. floridensis*, *L. fleischmannii*, and *L. aquatica*), which were unable to grow in BHI broth or on BHI agar at 4°C, a phenotype considered characteristic of the genus *Listeria* (27). Since then, five new *Listeria* species (*L. costaricensis*, *L. ilorinensis*, *L. goaensis*, *L. thailandensis*, and *L. valentina*), which cluster with *L. floridensis*, *L. fleischmannii*, and *L. aquatica*, have been identified. All these five newly described species share the inability to grow at 4°C, further supporting their re-classification, along with *L. floridensis*, *L. fleischmanniii*, and *L. aquatica* as a new genus named *Mesolisteria* (referring to the mesophilic nature of species within this genus). Notably, two proposed *Mesolisteria* species, *L. costaricensis* and *L. ilorinensis*, can be further differentiated from all currently described *Listeria* spp. by (i) the absence of catalase activity (catalase gene absent from the draft genomes), and (ii) atypical *Listeria* motility (motile only at 37°C, not at 20–25°C) (13, 18).

The other nine species within *Listeria sensu lato* cluster together in a clade, which has been suggested to represent another distinct genus, *Paenilisteria*. Members of this proposed genus show a number of phenotypic characteristics that distinguish them from *Listeria sensu stricto*. For example, all nine members of this clade are non-motile (while all *Listeria sensu stricto* species, except for *L. immobilis*, are motile) show negative results for the Voges-Proskauer test (while all *Listeria sensu stricto* show positive results for this test) and are able to reduce nitrate (while all *Listeria sensu stricto* are unable to reduce nitrate) (18). Hence, isolates of species in the proposed genus *Paenilisteria* may not be identified as *Listeria* with many confirmation procedures that target these phenotypes.

## ECOLOGY AND DISTRIBUTION

*Listeria* has been reported to inhabit a diverse array of environments, including soil, water, vegetation, sewage, animal feeds, farms, and food-processing settings (34–37). In the soil environment across the USA, *Listeria* has been reported to be particularly prevalent in and near the Mississippi River Basin, exhibiting an aggregated spatial distribution that is mainly governed by soil moisture, molybdenum, and salinity concentrations (20). In Austria, the presence of *Listeria* in environmental samples (soil and water) was found to be associated with lower altitudes with almost no *Listeria* being found at altitudes greater than 1,500 m (36). In addition, the texture of the soil, particularly its clay content, has been identified as a key factor affecting the long-term survival of *Listeria* in the soil environment (38). Overall, *Listeria* spp. exhibit heterogeneous distribution patterns, influenced by different environmental variables (39, 40). For example, Liao et al. (40) found that the two most important environmental variables for each species were precipitation and aluminum for *L. monocytogenes*, proximity to forest and wind speed for *L. seeligeri*, and annual maximum and minimum temperature and proximity to wetland for *L. innocua*, suggesting that *Listeria* species have distinct ecological preferences in the environment. The environmental factors influencing the distribution of *Listeria* also appear to vary based on the specific type of environment. For example, soil moisture and proximity to water and pastures were found to be associated with the presence of *Listeria* spp. in produce production environments in the state of New York, while elevation, study site (an undeveloped location in New York state, with minimal human presence, and that provided a habitat for wildlife [e.g., national forests and wildlife refuges]), and proximity to pastures were found to be associated with their presence in natural environments (41), suggesting that pastures and their associated grazing animals may be a common source of *Listeria* spp. contamination in agricultural and natural environments.

While the *Listeria sensu stricto* species described until 2020 (i.e., *L. monocytogenes*, *L. seeligeri*, *L. welshimeri*, *L. innocua*, *L. ivanovii*, and *L. marthii*) have been isolated from a variety of environments (36, 39, 42, 43) and appear to be globally distributed (except for *L. marthii*, which has only been isolated from natural and produce processing environments in the USA to date) (27, 43), the four new *sensu stricto* species described since 2021, *L. farberi*, *L. immobilis*, *L. cossartiae*, and *L. swaminathanii*, have been only isolated from soils in the natural environment in the USA to date (20). Compared to the classical *sensu stricto* species, especially *L. monocytogenes*, *L. seeligeri*, and *L. welshimeri*, these new species may be less prevalent in the environment; in one study, *L. farberi* was found in the southern USA, including Texas and Florida, while *L. immobilis* was detected in the northern USA, including the states of Wyoming, South Dakota, and Montana. Between the two subspecies of *L. cossartiae*, subsp. *cossartiae* was detected at multiple locations within the state of Alabama (USA), while subsp. *cayugensis*, although being isolated from fewer sites, was detected in two states, North Carolina and Georgia (USA). *L. swaminathanii* was detected at only one site in the Great Smoky Mountains with an elevation of 587 m in the state of North Carolina (USA) (20, 44). Overall, 5 of the 10 *Listeria sensu stricto* species have only been isolated in the USA, including *L. marthii*, which was first described in 2010, and four species (*L. cossartiae*, *L. farberi*, *L. immobilis*, and *L. swaminathanii*) that were described in 2022. Although these findings are based on very few studies and future studies involving in-depth sampling of natural environments in non-North America continents may find isolates from these five species in other continents, it is tempting to hypothesize that the common ancestor of the *Listeria sensu stricto* species originated in North America and that only those five species (*L. monocytogenes*, *L. innocua*, *L. seeligeri*, *L. ivanovii*, and *L. welshimeri*) that are commonly found associated with foods and food-associated environments have spread globally, probably following human migrations and transport of products across continents. In fact, previous studies using phylogeography suggested that *L. monocytogenes* Clonal Complex 1 (Lm-CC1), which is one of the most common and diverse CCs found today globally, originated in North America approximately 1,800 years ago and then spread worldwide starting in the 19th century (45, 46).

All 17 *Listeria sensu lato* species, except for *L. rocourtiae* and *L. grayi*, were described between 2013 and 2022, and the ecology of the *sensu lato* species is hence less well understood. To date, only one *sensu lato* species, *L. grayi*, has been isolated in Europe, Asia, Africa, South America, and North America (27). There is, however, evidence that some of the more recently discovered *sensu lato* species are also widely distributed. For example, after its discovery in 2015, *L. booriae* has been isolated from 67 locations across 21 U.S. states, including North Carolina, South Carolina, Texas, Ohio, Alabama, Kentucky, New York, Pennsylvania, Maryland, Indiana, Georgia, Illinois, Minnesota, North Dakota, South Dakota, Wyoming, Tennessee, Montana, Florida, Mississippi, and Louisiana (20). This wide habitat breadth of *L. booriae* in the USA indicates its ability to survive in a wide range of environmental conditions and suggests that *L. booriae* may be prevalent in other countries as well, especially in soils. Two *sensu lato* species, *L. newyorkensis* and *L. fleischmannii*, have been isolated from both natural environments and food or food-associated environments in both North America and Europe. *L. newyorkensis* has been detected in soils in the state of Minnesota, USA (20), raw milk in Italy (47), river water in Japan (48), and in a seafood processing facility in northeastern USA (12). *L. fleischmannii* has been isolated from soils in the state of Iowa, USA (20), cheeses in Italy and Switzerland (10), and the environment of a cattle ranch in the state of Colorado, USA (49). Both *L. grandensis* and *L. aquatica* have been found in both soil and water environments in the USA. *L. grandensis* has been detected in soils in the states of Minnesota, Wyoming, and Montana (20) and water in the state of Colorado (11), while *L. aquatica* has been found in soils in the state of Utah (11) and water in the state of Florida (20). Since first being isolated from fried chicken in Thailand (15), *L. thailandensis* has also been identified in South Africa (50). *L. valentina* was isolated from a water trough and the feces of healthy sheep in an animal farm environment in Valencia, Spain (16). The remaining *sensu lato* species, however, have only been isolated from a few samples and locations. While future studies are needed to define the ecological niches of the newly identified *Listeria sensu lato* species, our current limited data may suggest that some *Listeria sensu lato* species may inhabit more specialized ecological niches and/or may be less widely distributed. For example, *L. floridensis*, *L. riparia*, and *L. cornellensis* have, so far, only been isolated from aquatic environments in the USA (11). *L. floridensis* and *L. riparia* were found in the state of Florida (11), while *L. cornellensis* was found in the state of Colorado (11). *L. portnoyi* and *L. rustica* have been exclusively found in soils in the natural environment in the state of South Dakota, USA, and agricultural water in the state of New York, USA, respectively (17). *L. rocourtiae*, *L. weihenstephanensis*, *L. costaricensis*, *L. goaensis*, and the most recently characterized *sensu lato* species, *L. ilorinensis*, have been isolated, so far, in Austria, Germany, Costa Rica, India, and Nigeria, respectively. Specifically, *L. rocourtiae* was isolated from a sample of pre-cut lettuce in Austria, *L. weihenstephanensis* was isolated from the water plant *Lemna trisulca* from a pond in Germany, and *L. costaricensis* was isolated from a food processing drainage system in Costa Rica (13). *L. goaensis* was isolated from mangrove swamps in Goa, India (14), and *L. ilorinensis* was isolated from cow milk cheese in Nigeria (18). Hence, *Listeria sensu lato* newly described species have been isolated from around the world and from different environment types, including many species only isolated from natural environments.

## DETECTION AND IDENTIFICATION CHALLENGES

Detection and identification of *Listeria* species can be performed using either conventional (“classic”) methods (e.g., selective enrichment and plating) or rapid methods (e.g., immunoassays and DNA-based assays such as PCR). For detection using conventional methods, a *Listeria* species must (i) grow to detectable levels in selective enrichment broth and (ii) possess specific characteristics targeted by the method (e.g., motility, colony morphology, and color on differential media). For detection using rapid methods, a *Listeria* species must (i) grow to detectable levels in selective enrichment broth and (ii) possess specific characteristics targeted by the method (e.g., the presence of specific antigens, such as flagella, or the presence of a target gene). Finally, confirmation procedures that characterize putative *Listeria* colonies or isolates are an essential part of many detection protocols for both conventional and rapid methods; common confirmation procedures are either multiplexed phenotypic tests (e.g., analytical profile index strips), multiplex PCR tests that target specific DNA markers, or matrix-assisted laser desorption/ionization time-of-flight mass spectrometry methods that generates a proteomic fingerprint pattern for an isolate to determine its species classification (51, 52). In some cases, these comprehensive characterization procedures are only indicated if isolates show *Listeria*-like characteristics with specific initial screening tests (e.g., motility).

Importantly, for some applications, testing focuses on the detection of all *Listeria* species (e.g., as part of environmental monitoring of food processing facilities), while in other cases, specific testing for *L. monocytogenes* is performed. The pace of the *Listeria* genus expansion has exceeded the pace conventional, and rapid methods can be revised and has revealed a number of challenges for both *Listeria* spp. and *L. monocytogenes* detection, as well as confirmation procedures (Table 1). Importantly, recent studies have shown that (i) some *sensu lato* species will not be detectable with at least some conventional detection method, including due to their inability to grow to detectable levels in some enrichment media; (ii) some *Listeria sensu lato* species could potentially outgrow certain “classic” *Listeria* in some enrichment media (29), presenting a risk for false negative results with conventional or rapid methods; (iii) most rapid methods will likely only detect *Listeria sensu stricto* species (53); (iv) some of the recently discovered *Listeria* spp. (i.e., *L. marthii*, *L. cossartiae*, and *L. swaminathanii*) could yield false positive results for *L. monocytogenes* with rapid methods that do not target virulence genes/factors (19); and (v) confirmation methods may misidentify a number of the recently identified *Listeria* species, and of particular concern, may misidentify, as *L. monocytogenes*, some *Listeria* spp. closely related to *L. monocytogenes*.

**TABLE 1:** Differentiating characteristics and challenges associated with the detection and identification of *Listeria* species and clades

Species/group	Differentiating and notable characteristics	Challenges associated with:		
		Conventional detection methods	Rapid detection methods	Identification methods
*Listeria sensu stricto clade*				
*L. monocytogenes*	β-Hemolytic, PI-PLC positive, positive rhamnose acidification, and α-mannosidase activity	None predicted	None predicted	None predicted
*L. innocua*	PI-PLC negative, positive α-arylamidase activity, and α-mannosidase activity	None predicted	Hemolytic *L. innocua* may yield false positives with some *L. monocytogenes* PCR methods	Hemolytic *L. innocua* may be mis-identified as *L. monocytogenes*
*L. ivanovii*	β-Hemolytic and PI-PLC positive	Grows poorly in some selective enrichment media (may be outgrown by some *Listeria senso lato* in certain enrichment media)^*a*^ and may show atypical colonies on some plating media	None predicted	None predicted
*L. welshimeri*	Positive tagatose acidification	None predicted	None predicted	None predicted
*L. immobilis*	Non-motile	None predicted	No detection by immunoassays that target flagellar antigens (leading to false negatives)	May not be identified correctly as *L. immobilis* (as databases for identification methods may not have been updated)
*L. marthii*, *L. cossartiae*, and *L swaminathanii*	Non-hemolytic, PI-PLC negative, negative rhamnose acidification, and negative α-arylamidase activity	None predicted	May be detected by methods designed to specifically detect *L. monocytogenes* (false positives) if virulence genes/factors are not targeted by the method	May be misidentified as *L. monocytogenes* if virulence genes/factors are not characterized by the method or may be misidentified as another species (as databases for identification methods may not have been updated)
*L seeligeri*	Weakly β-hemolytic	Grows poorly in some selective enrichment media (may be outgrown by some *Listeria senso lato* in certain enrichment media)^*a*^ and may show atypical colonies on some plating media	None predicted	None predicted
*Murraya* clade				
*L. grayi*	Positive mannitol acidification and motile	Grows poorly in some enrichment media and may show atypical colonies on some plating media (both issues may lead to possible false negatives)	May grow poorly in some enrichment media (leading to possible false negatives)	None predicted
*Mesolisteria* clade^*b*^				
*L. floridensis*	Unable to grow at 4°C, non-motile	Grows poorly in some selective enrichment media (may lead to possible false negatives)	Will not be detected by immunoassay that target flagellar antigens	Most databases will not be able to identify at the species level
*L. costaricensis*	Unable to grow at 4°C, catalase-negative, and motility observed only at 37°C	Grows poorly in some selective enrichment media (may lead to possible false negatives)	May not be detected by immunoassay that target flagellar antigens	Most databases will not be able to identify at the species level
*L. ilorinensis*	Unable to grow at 4°C, catalase-negative, and motility observed only at 37°C	Not assessed	May not be detected by immunoassay that target flagellar antigens	Most databases will not be able to identify at the species level
*L. fleischmannii, L. aquatica, L. valentina, and L. thailandensis*	Unable to grow at 4°C, non-motile, and reduces nitrate	None predicted	Will not be detected by immunoassay that target flagellar antigens	Most databases will not be able to identify at the species level
*L. goaensis*	Unable to grow at 4°C and non-motile	None predicted	Will not be detected by immunoassay that target flagellar antigens	Most databases will not be able to identify at the species level
*Paenilisteria* clade				
*L. rocourtiae*	Reduces nitrate and non-motile	May grow poorly in some selective enrichment media	Will not be detected by immunoassay that target flagellar antigens	Most databases will not be able to identify at the species level
*L. cornellensis*, *L. grandensis, L. weihenstephanensis*, *L. rustica^c^*, and *L. portnoyi^c^*	Reduces nitrate and non-motile	May grow poorly in some selective enrichment media and may show atypical colonies on some plating media	Will not be detected by immunoassay that target flagellar antigens	Most databases will not be able to identify at the species level

^*a*^ This issue may also occur with other species, but further experiments will be required to determine this.

^*b*^ *L. valentina* and *L. ilorinensis* selective enrichment media growth and rapid detection data not available. *L. costaricensis* was detected by immunoassays targeting flagellar antigens.

^*c*^ *L. portnoyi* and *L. rustica* growth have not been evaluated in selective media; these species yielded no growth and reduced growth, respectively, from nonselective media (BHI) incubated at 37°C.

### Detection using conventional methods

Given the genotypic and phenotypic similarity of the four newly described *Listeria sensu stricto* species (*L. cossartiae*, *L. farberi*, *L. immobilis* [17], and *L. swaminathanii* [19, 26]) to the six species described prior to 2021 (*L. monocytogenes*, *L. innocua*, *L. ivanovii*, *L. seeligeri*, *L. welshimeri*, and *L. marthii*), it is expected that the newly described *sensu stricto* species will grow to detectable levels and be detected by the currently available conventional detection methods for *Listeria* spp. (although more experimental work will be needed to confirm this supposition). Conversely, it has been clearly documented that *Listeria sensu lato* species differ tremendously in their ability to grow in different selective enrichment media (29). Specifically, several *Listeria sensu lato* species (e.g., *L. cornellensis* and *L. grandensis*) may not grow to detectable levels in certain enrichment media, such as Fraser and UVM (29, 54); some *sensu lato* species thus may not easily be detectable with at least some conventional detection methods, and future studies will be needed to identify the reason for this. On the other hand, some *Listeria sensu lato* species (e.g., *L. booriae* and *L. thailandensis*) may grow to high levels and possibly outgrow a *Listeria sensu stricto* (e.g., *L. seeligeri*) (29). This can present a risk for false negative results, particularly with rapid detection methods that may be able to yield positive results with *Listeria sensu stricto*, but not with *sensu lato* species (e.g., immunoassays that target flagella as detailed below).

Our current knowledge of the growth patterns of different *Listeria* species also suggests the potential for false negative results with conventional methods for *L. monocytogenes* as some *Listeria* species may outgrow *L. monocytogenes* during enrichment (as previously reported for *L. innocua* [55–57]). This would be a particular concern if conventional methods are used that do not include differential plating and rather rely on speciation of an often small number of colonies from selective plating media for *L. monocytogenes* detection. While this issue can be mitigated with the use of differential plating media (e.g., ALOA), these media may not be easily accessible in all countries. Further co-inoculation studies with both *L. monocytogenes* and other *Listeria* spp. will, however, be needed to better characterize the risk of false negative *L. monocytogenes* results due to competition during different enrichment procedures.

### Detection using rapid methods

The identification and characterization of new *Listeria* spp. also has led to new insights regarding the ability of different rapid methods to comprehensively detect all current *Listeria* spp. (i.e., both *sensu lato* and *sensu stricto*). Specifically, a study evaluating the detection of the recently described *Listeria* species (53) supports that the currently available *Listeria* spp. rapid detection methods, while not validated with the newly described *Listeria sensu stricto* species, will likely detect all five newly described *sensu stricto* species. The only exception is *L. immobilis*, which will not be detected by immunoassays that target flagellar proteins (53); this species is non-motile and lacks all genes in the flagella locus (17). On the other hand, currently available rapid methods are unlikely to detect all *Listeria sensu lato* species (53). More specifically, many immunoassays are likely to not detect a number of *Listeria sensu lato* species as 15/18 species in this group lack flagellar motility and flagellar antigens are frequently targeted by *Listeria* immunoassays. With regard to DNA-based detection methods, *sensu lato* groups likely represent a challenge as they are generally genetically very distinct from the classical *Listeria* species, which were used for the development and validation of essentially all methods in the market as of 2023. Future work is, however, needed to assess different DNA-based methods for the ability to detect *sensu lato* species; this validation could be performed *in silico* (e.g., by assessing primer and probe sequences for their homology to target gene sequence found in these species) or experimentally.

With regard to detection of *L. monocytogenes* by rapid methods, rapid methods that target virulence genes (e.g., *hly*) or virulence factors are not expected to cause false positive results with any of the new *Listeria* spp.; these assays, however, may cause what could be considered false positive with hemolytic *L. innocua* (i.e., *L. innocua* strains that carry orthologs of the major virulence genes found in *L. monocytogenes* and *L. ivanovii* [58]). However, molecular methods for *L. monocytogenes* that target non-virulence genes, which were typically developed based on the classical *Listeria* species, may cause false positive for *L. monocytogenes* with *sensu stricto* species that are closely related to *L. monocytogenes* (i.e., *L. marthii*, *L. cossartiae*, and *L. swaminathanii*), supported by the fact that the now discontinued Accuprobe *L. monocytogenes* test yielded false positive results with *L. marthii* (7). Further work is thus needed to assess at least some *L. monocytogenes* detection methods (i.e., those targeting non-virulence genes) for the risk of false positive results with *Listeria* spp. closely related to *L. monocytogenes*.

### Identification procedures

The discovery of new *Listeria* species also has revealed challenges with identification methods, which are typically used to (i) confirm the presence or isolation of *Listeria* species (i.e., by differentiating them from other species that show similar phenotypes, such as on MOX plates) or to (ii) classify colonies or pure cultures to the species level. Challenges can occur with individual information assays (e.g., motility assays) as well as multiplexed phenotypic assays (e.g., API) or mass spec-based characterization methods. For example, the discovery of a number of non-motile *Listeria* species indicates that the use of motility assay as a primary screen to determine whether a given colony represents a *Listeria* would lead to a number of false negatives, which should be clarified across standard methods that still recommend a motility assay. For multiplex phenotypic and mass-spec-based identification methods, a key challenge is that the databases utilized by these systems often do not include data for many of the recently discovered *Listeria* species. For example, among the commonly used commercial identification methods, only the Bruker Biotyper (59) contains a selection (8 out of 17) of the recently described *Listeria sensu lato*. Hence, with many identification methods a number of the newly described *Listeria* species may yield either (i) no possible match, (ii) an identification to the genus level, or (iii) misidentification of the species. For example, a *Listeria sensu lato* species that (i) utilizes xylose, (ii) does not utilize rhamnose or mannitol, and (ii) is negative for α-mannosidase activity could be misidentified as the *sensu stricto L. ivanovii* (18) unless the organism is also tested for β-hemolysis and PI-PLC activity (tests that are positive for *L. ivanovii* and negative for all *Listeria sensu lato*). As whole-genome sequencing (WGS) becomes cheaper and faster with advances in technology, WGS of colonies obtained from selective media (e.g., MOX) could be used for species identification using scientifically recognizable objective criteria, such as an average nucleotide identity of 95% for species identification (60).

Importantly, an incomplete database may also lead to the misidentification of non-pathogenic *Listeria* spp. as *L. monocytogenes*. For example, *L. marthii* shows high similarity to *L. monocytogenes* (7) and is often not represented in rapid identification method databases. Therefore, species that are similar to *L. marthii* (and *L. marthii* itself) could be misidentified as *L. monocytogenes*. Currently, among commonly used identification methods, only the VITEK MS database includes *L. marthii* (61). Notably, none of the newly described *sensu stricto* species that are closely related to *L. monocytogenes* and may likely be misidentified as *L. monocytogenes* (i.e. *L. marthii*, *L. cossartiae*, and *L. swaminathanii*) are pathogenic and their genomes lack the pathogenic characteristics targeted by most *L. monocytogenes* detection methods (e.g., identification of the hemolysin gene, *hly* [62–64]); therefore, these species are not expected to yield *L. monocytogenes* false positives with an identification method that include tests that targets virulence genes. An easy way to avoid misclassification of a non-pathogenic *Listeria* as *L. monocytogenes* would thus be to also perform either (i) a PCR screen for one or more virulence genes; (ii) a screen for virulence factor activity (e.g., RAPID´L.mono medium, which differentiates *Listeria* colonies based on PI-PLC activity, ß-hemolysis assay using blood agar); or (iii) WGS.

## IMPLICATIONS FOR FOOD SAFETY

The discovery of five new *Listeria sensu stricto* species as well as 17 additional new *Listeria sensu lato* species since 2010 has a number of implications for food safety, including (i) clearly identified risks for false positive test results for *L. monocytogenes*; (ii) potential for false negative test results for *L. monocytogenes*; and (iii) risk of false negative results for *Listeria* spp. In addition, the discovery, genomic, and phenotypic characterization of *Listeria sensu lato* species raises the issue of whether all members of the *Listeria sensu lato* genus really should be considered “index” or “indicator” organisms, as well as which species would make appropriate surrogates (for example for use in inoculation studies).

Importantly, newly discovered *Listeria sensu stricto* species that are closely related to *L. monocytogenes* (e.g., *L. cossartiae* and *L. swaminathanii*) can generate false positive results with rapid DNA or antigen-based methods and specifically those methods that target molecules that are not *L. monocytogenes* specific virulence factors; a re-evaluation and possibly revalidation of *L. monocytogenes* specific rapid detection methods that target non-virulence related targets may thus be needed. In addition, confirmation of rapid method positives for *L. monocytogenes* can help identify false positive results due to the presence of *Listeria* spp. closely related to *L. monocytogenes*. It is, however, essential to assure that confirmation methods can differentiate *L. monocytogenes* from closely related *Listeria* species; challenges can arise if methods and databases used for result interpretation have not been updated to include all newly identified *Listeria* spp. closely related to *L. monocytogenes*. WGS-based confirmation of *L. monocytogenes* positives will provide the most reliable confirmation approach, with the possibility of using shotgun sequencing to directly recover genetic materials from enrichment cultures (sometimes referred to as “quasi-metagenomics” [65]) as these approaches are being further developed and validated. While specific detection of *L. monocytogenes* specific virulence genes (e.g., *hly*) using PCR-based or other molecular methods offers another option for confirmation, these approaches cannot differentiate between *L. monocytogenes* and hemolytic *L. innocua* and may thus be less valuable. While future possible discovery of additional *Listeria* spp. closely related to *L. monocytogenes* may, unfortunately, require additional re-evaluation of detection methods, this issue is not limited to *Listeria* and may apply to detection methods for other pathogens.

The identification and characterization of newly discovered *Listeria sensu stricto* and *sensu lato* species have also helped to define possible scenarios with an increased risk of false negative results for the *sensu stricto* species, which represent good indicators of condition in which *L. monocytogenes* can thrive. This risk of obtaining false negatives for *L. monocytogenes* indicators is supported by experimental data (29) that suggest that some *Listeria sensu lato* species that cannot grow at low temperatures (e.g. *L. fleischmannii*, *L. floridensis*, and *L. costaricensis*) and, therefore, are not good indicators of conditions where *L. monocytogenes* can thrive and may grow to significantly higher levels than *L. ivanovii* and *L. seeligeri* (both considered good indicators for *L. monocytogenes*). These findings raise concerns that *L. ivanovii* (or *L. seeligeri*) could be detected by a rapid method, but not isolated in the culture confirmation, if a given sample contains both *L. ivanovii* as well as a *Listeria sensu lato* species that grows faster than *L. ivanovii*. Further co-inoculation experiments will, however, need to be performed to further test this hypothesis; if inter-genus competition is confirmed as an issue that could cause false negative results, re-design and improvement of enrichment procedures may be needed in the long term to address this issue.

Other scenarios may also lead to increased risks of false negative results for *Listeria* spp. using rapid identification methods. In particular, false negative results may occur with *Listeria sensu lato* strains that are both distinct from *L. monocytogenes* and from the “classical” *Listeria* species (e.g., *L. welshimeri* and *L. seeligeri*) as currently available rapid methods typically were neither designed nor validated to detect these types of newly described *Listeria* spp. Future efforts are thus needed to assess methods for the ability to detect and identify all currently described *Listeria* species, which will be needed to include efforts to update databases and manuals to include phenotypic data for all *Listeria* spp. While, as detailed in the next paragraph, the inability of a given method or assay to detect some or all *Listeria sensu lato* species may not be a concern, information on the inclusivity of different *Listeria* spp. detection methods and assays should be provided and up to date with information for all *Listeria* spp.

A final challenge that has become apparent with the recent description of 17 *Listeria sensu lato* species relates to the use of rapid methods that detect *Listeria* spp. to identify conditions, in food-processing facilities, that increase the risk of *L. monocytogenes* presence, growth, and/or contamination and the use of this information to reduce food contamination with *L. monocytogenes* and hence human listeriosis cases (i.e., the use of *Listeria* species as index or indicator organisms). With phenotypic data that show that *Listeria sensu lato* species have characteristics that are distinct from *L. monocytogenes* to such an extent that these differences suggest different habitats as well as survival and growth capabilities (e.g., the fact that *Listeria sensu lato* species proposed to be renamed as “*Mesolisteria*” do not grow in BHI broth or BHI agar at refrigeration temperatures), it has become clear that the detection of the *Listeria* species grouped into *Listeria sensu lato* does not indicate conditions that suggest an increased risk of *L. monocytogenes* presence, growth, and/or contamination. The creation of three new genera, as supported by the distinct genomic and phenotypic characteristics of the three proposed new genera that represent the current *Listeria sensu lato* (as detailed above), would resolve this issue and provide industry with a clear direction to focus testing on the current *Listeria sensu stricto* species, which represent appropriate indicator/index organisms for conditions that may increase the risk of *L. monocytogenes* presence. Alternatively, but more difficult and possibly confusing, industry could select *Listeria* spp. assays and detection methods that specifically detect *Listeria sensu stricto* species and do not detect *Listeria sensu lato*. Regardless of which path is selected short and long term, increased education of industry and regulatory agencies on the different *Listeria* species and their value as index or indicator organisms is needed. It is important to also note that continued discovery of additional new species is likely, particularly if increased *Listeria* surveillance and testing are performed in regions, habitats, foods, and food ingredients that have not been previously surveyed for *Listeria* spp.; as new species are identified, further re-evaluation of detection methods and taxonomy thus may be needed.

## SUPPLEMENTAL MATERIAL

Table S1 mbio.00938-23-s0001.docx Biochemical characteristics and carbohydrate acidification profiles for the differentiation of *Listeria* species.

ASM does not own the copyrights to Supplemental Material that may be linked to, or accessed through, an article. The authors have granted ASM a non-exclusive, world-wide license to publish the Supplemental Material files. Please contact the corresponding author directly for reuse.
