# Supplementary material for: Taxonomy, ecology, and relevance to food safety of the genus Listeria with a particular consideration of new Listeria species described between 2010 and 2022
Source: mBio. 2023 Dec 21;15(2):e00938-23. doi: 10.1128/mbio.00938-23 (PMC10865800; doi:10.1128/mbio.00938-23)
Supplement: Table S1 — Biochemical characteristics and carbohydrate acidification profiles for the differentiation of Listeria species. [file mbio.00938-23-s0001.docx]

|  | Hemolytic | PI-PLC^1^ | DIM^2^ | αMAN^3^ | Mannitol | Rhamnose | Xylose | Tagatose | Nitrate Reduction | Motility | Growth at 4°C | Catalase |
| --- | --- | --- | --- | --- | --- | --- | --- | --- | --- | --- | --- | --- |
| *Listeria sensu stricto* |  |  |  |  |  |  |  |  |  |  |  |  |
| *L. monocytogenes* | + | + | - | + | - | + | - | - | - | + | + | + |
| *L. innocua* | - | - | + | + | - | v | - | - | - | + | + | + |
| *L. seeligeri* | (+) | - | + | - | - | - | + | - | - | + | + | + |
| *L. welshimeri* | - | - | v | + | - | v | + | + | - | + | + | + |
| *L. ivanovii* | + | + | v | - | - | - | + | - | - | + | + | + |
| *L. marthii* | - | - | - | + | - | - | - | - | - | + | + | + |
| *L. farberi* | - | - | + | + | - | + | - | - | - | + | + | + |
| *L. immobilis* | - | - | - | - | - | - | + | - | - | - | + | + |
| *L. cossartiae* | - | - | - | + | - | - | - | - | - | + | + | + |
| *L. swaminathanii* | - | - | - | + | - | - | - | - | - | + | + | + |
| *Listeria sensu lato* |  |  |  |  |  |  |  |  |  |  |  |  |
| *Murraya* |  |  |  |  |  |  |  |  |  |  |  |  |
| *L. grayi* | - | - | + | v | + | v | - | - | v | + | + | + |
| *Paenelisteria* |  |  |  |  |  |  |  |  |  |  |  |  |
| *L. rustica* | - | - | - | - | + | + | + | - | + | - | + | + |
| *L. portnoyi* | - | - | - | - | + | + | + | - | + | - | + | + |
| *L. cornellensis* | - | - | - | - | - | - | + | - | + | - | + | + |
| *L. newyorkensis* | - | - | - | - | + | + | + | - | + | - | + | + |
| *L. rocourtiae* | - | - | - | + | + | + | + | - | + | - | + | + |
| *L. weihenstephanensis* | - | - | - |  | + | + | + | - | + | - | + | + |
| *L. grandensis* | - | - | - |  | - | - | + | - | + | - | + | + |
| *L. booriae* | - | - | - | + | + | + | + | v | + | - | + | + |
| *L. riparia* | - | - | - | + | + | v | + | - | + | - | + | + |
| *Mesolisteria* |  |  |  |  |  |  |  |  |  |  |  |  |
| *L. fleischmannii* | - | - | - | - | v | v | + | - | + | - | - | + |
| *L. floridensis* | - | - | - | - | - | - | + | - | - | - | - | + |
| *L. aquatica* | - | - | - | + | - | - | + | + | + | - | - | + |
| *L. valentina* | - | - | - | - | - | - | + | v | + | - | - | + |
| *L. thailandensis* | - | - | - | - |  | - | + | + | + | - | - | + |
| *L. goaensis* | - | - | - | - | - | - | + | - | - | - | - | + |
| *L. ilorinensis* | - | - | - | - | - | - | + | - | - | +(37°C)^4^ | - | - |
| *L. costaricensis* | - | - | - | - | - | - | + | - | - | +(37°C) | - | - |

**Supplemental Table 1**: Biochemical characteristics and carbohydrate acidification profiles for the differentiation of *Listeria* species

+, positive; -, negative; (+), weakly positive; v, variable; ^1^PI-PLC, Phosphoinositide phospholipase C; ^2^DIM, Differentiation/Innocua/Moncytogenes test that is part of the API *Listeria* (bioMérieux) and detects α-arylamidase activity,^3^αMAN, α-mannosidase activity, ^4^Motility is only observed at 37˚C
